# A Sequential Multiplicative Extended Kalman Filter for Attitude Estimation Using Vector Observations

**DOI:** 10.3390/s18051414

**Published:** 2018-05-03

**Authors:** Fangjun Qin, Lubin Chang, Sai Jiang, Feng Zha

**Affiliations:** 1 Department of Navigation Engineering, Naval University of Engineering, Wuhan 430000, China; haig2005@126.com (F.Q.); zha_feng@126.com (F.Z.); 2 Office of Research and Development, Naval University of Engineering, Wuhan 430000, China; jsqfj@126.com

**Keywords:** attitude estimation, multiplicative extended Kalman filter, sequential estimation

## Abstract

In this paper, a sequential multiplicative extended Kalman filter (SMEKF) is proposed for attitude estimation using vector observations. In the proposed SMEKF, each of the vector observations is processed sequentially to update the attitude, which can make the measurement model linearization more accurate for the next vector observation. This is the main difference to Murrell’s variation of the MEKF, which does not update the attitude estimate during the sequential procedure. Meanwhile, the covariance is updated after all the vector observations have been processed, which is used to account for the special characteristics of the reset operation necessary for the attitude update. This is the main difference to the traditional sequential EKF, which updates the state covariance at each step of the sequential procedure. The numerical simulation study demonstrates that the proposed SMEKF has more consistent and accurate performance in a wide range of initial estimate errors compared to the MEKF and its traditional sequential forms.

## 1. Introduction

A spacecraft’s attitude information is necessary for most space missions, and is often derived from attitude estimation algorithms using onboard sensor data [1,2]. The applicable sensors include *star trackers, sun sensors, horizon sensors, magnetometers, global positioning systems* and so on. Attitude estimation, in general, refers to some filtering-based approaches that take a dynamic model of the spacecraft’s motion into account [2]. Generally, research into attitude estimation centers principally on two aspects: attitude parameterization and filtering algorithms. As for the aspect of attitude parameterization, researchers and engineers have been recognizing the utility of employing quaternion due to its computational efficiency and singularity avoidance. For attitude estimation filtering algorithms, Kalman type filters have received the most attention due in part to their ease of implementation and modest computational cost. However, the primary issue with the implementation of Kalman type filters in attitude estimation is maintaining the quaternion unit norm. A mathematically elegant solution to this problem is to use the quaternion for global attitude representation, taking singularity avoidance into consideration, while using unconstrained parameters for local attitude representation in the filtering process with reduced rank covariance [3]. Within this framework, many general filtering algorithms have been applied to the attitude estimation problem, such as extended Kalman filter (EKF), unscented Kalman filter (UKF), cubature Kalman filter (CKF), divided difference filter (DDF), and so on [3,4,5,6,7,8]. For more details on these attitude estimation algorithms, one can refer to the survey paper [9] and the most recent monograph [2]. Among these filtering algorithms, the most representative are the EKF in its multiplicative form known as multiplicative EKF (MEKF) and UKF in its quaternion form known as unscented quaternion estimator (USQUE). Although the USQUE will be more accurate when a good priori estimate of the attitude is unavailable, its computational burden is cumbersome. Up to now, the MEKF is still the most prolific approach to on-board attitude estimation. Although considerable performance degradation may arise from the implicit linearization in MEKF when large initial condition errors are involved, some strategies within the MEKF framework can still be used to circumvent this drawback. This paper is dedicated to improving the performance of the MEKF taking both accuracy and computational efficiency into consideration.

Generally, spacecraft can obtain multiple or/and many noisy measurements in vector observations formed simultaneously from different attitude sensors, such as a three-axis magnetometer, star tracker, sun sensor, and so on. In this case, the Kalman gain necessitates an inversion of a 3n×3n matrix with n denoting the number of vector observations. [1] argues that this inversion is a cumbersome computational burden for real-time applications and a Murrell’s variation of the MEKF (MMEKF) was developed to avoid this expensive computation based on the linear update structure of the Kalman type filter (KTF). That is, the MMEKF was originally developed to alleviate the computational costs of the MEKF and cannot improve the accuracy performance of the MEKF. Murrell’s variation is virtually a sequential processing scheme of the measurements obtained simultaneously. In fact, the sequential strategy can also be used to improve the filtering performance in terms of accuracy. In [10,11,12], the sequential processing scheme is applied to EKF-based radar target tracking problems, resulting in a filtering algorithm favorable to both estimation accuracy and computational efficiency. However, the sequential structure in [10,11,12] cannot be transplanted to the MEKF superficially due to the special structure of the MEKF and the special characteristics of the attitude. These aforementioned facts represent the main motivation of this paper, which will focus on the investigation of a sequential processing scheme for the MEKF for attitude estimation. In the proposed sequential MEKF (SMEKF), the single vector observation model is re-linearized regarding the updated state estimate when the last single vector observation has been processed. This is the manner in which the proposed SMEKF can improve the accuracy performance, and is the main difference to the MMEKF. Meanwhile, in the sequential processing of these vector observations, the covariance is unchanged due to the special reset operation of the MEKF. This is the main difference to the traditional sequential EKF (SEKF).

The remainder of this paper is organized as follows. The MEKF and MMEKF for attitude estimation is first presented in Section 2 to provide the necessary background information. In Section 3, the SMEKF is developed in detail and compared to the MMEKF and the traditional SEKF. Finally, the numerical simulation results are reported alongside some traditional attitude estimation algorithms to illustrate the validity and superiority of the proposed SMEKF.

This paper can be viewed as a companion work to [13], which makes use of iteration procedure to improve the performance of the MEKF. This paper and [13] both seek to improve the filtering performance of some classical strategies within the MEKF framework. The main contribution of these works lies in their examination of the ways in which the classical strategies can be integrated into the special structure of MEKF.

## 2. MEKF and MMEKF

The attitude estimation problem with the quaternion kinematics model is given by

(1) $$\dot{q}=\frac{1}{2}\Xi \left(q\right)\omega$$

where the quaternion $q={\left[\rho^{T},q_{4}\right]}^{T}$ must obey a normalization constraint $q^{T}q=1$.

(2) $$\Xi \left(q\right)=\left[\begin{array}{c} q_{4}I_{3\times 3}+\left[\rho \times \right]\\ -\rho^{T} \end{array}\right]$$

where $I_{3\times 3}$ is a 3 × 3 identity matrix and [ρ×] is the cross-product matrix.

The angular rate ω is measured by a rate-integrating gyro, whose model is given by

(3) $$\begin{array}{l} \omega =\tilde{\omega }-\beta -\eta_{v}\\ \dot{\beta }=\eta_{u} \end{array}$$

where $\tilde{\omega }$ is the measured observation and β is a bias vector. $\eta_{v}$ and $\eta_{u}$ are zero-mean Gaussian white noise processes with covariances usually given by $\sigma_{v}^{2}I_{3\times 3}$ and $\sigma_{u}^{2}I_{3\times 3}$.

The discrete-time measurement function with *n* vector observations is given by

(4) $${\tilde{y}}_{k}={\left[\begin{array}{c} A\left(q\right)r_{1}\\ A\left(q\right)r_{2}\\ \vdots \\ A\left(q\right)r_{n} \end{array}\right]|}_{t_{k}}+{\left[\begin{array}{c} \upsilon_{1}\\ \upsilon_{2}\\ \vdots \\ \upsilon_{n} \end{array}\right]|}_{t_{k}}$$

In (4), A(q) is the rotation matrix corresponding to the quaternion q. $\upsilon_{i}$ is the measurement white noise for the *i*-th vector observation, whose covariance is $\sigma_{i}^{2}I_{3\times 3}$. In this respect, the measurement noise is given by $R=diag\left[\sigma_{1}^{2}I_{3\times 3}\;\sigma_{2}^{2}I_{3\times 3}\cdots \sigma_{n}^{2}I_{3\times 3}\right]$.

The explicit MEKF and MMEKF for attitude estimation are summarized in Table 1.

**Remark** **1.** *It can be seen from Table 1 that the only difference between MEKF and MMEKF lies in the measurement update. In the MMEKF, the measurement update is performed for each vector observation individually. According to the gain calculation, Murrell’s approach reduces the taking of the inverse of a* 3n×3n *matrix to taking the inverse of a* 3×3 *matrix. At first glance, this seems to significantly decrease the computational load. However, many other computational cost equations have also been added with Murrell’s variation. Therefore, the actual computational savings obtained by Murrell’s approach are not straightforward, which will be shown in the simulation study.*

**Remark** **2.** *It has been shown that during each processing of the vector observation, the measurement model is always linearized about the prior attitude estimate. This is the reason why Murrell’s approach cannot improve the accuracy performance.*

**Remark** **3.** *There is an explicit reset for the error state before the measurement update loop of the MMEKF, that is* $\Delta {\hat{x}}_{k}=0_{6\times 1}$ *. The reset performed before the measurement update loop can also be viewed as being performed before the time update loop. This is because that the priori error state remains the zero vector in the time update. Actually, the reset operation has also been performed implicitly in the MEKF.This can be deduced from the state update equation* $\Delta {\hat{x}}_{k}=K_{k}\left[{\tilde{y}}_{k}-h_{k}^{}\left({\hat{x}}_{k|k-1}\right)\right]$ *. This reset operation is used to move the update information from the error state to the full state estimate [4].*

## 3. SMEKF

In this section, the SMEKF is firstly presented explicitly. Some discussion of the proposed SMEKF is then presented with the main focus on the differences to the MMEKF and the general SEKF.

The explicit SMEKF for attitude estimation is summarized in Table 2. Although the time update of the SMEKF is the same as that of the MEKF, it is still presented for completeness.

**Remark** **4.** *Table 2 shows that the measurement update and attitude update are merged together into the sequential processing loop. That is, the posteriori error state is set to the zero vector after each sequential measurement update, reflecting that the expansions in the linearization of the next vector observation equation are now performed for the new state estimate. Since the posteriori state estimate (especially the attitude quaternion estimate) is more accurate than the prior estimate, the resulting sequential algorithm will outperform the MEKF and MMEKF in terms of accuracy.*

**Remark** **5.** *Assume that any of these measured vector observations is given by* $v_{B}$ *and its corresponding reference vector is given by* $v_{I}$ *. Their relationship is given by*

(5) $$\begin{array}{l} v_{B}=A\left(q\right)v_{I}=A\left(q\left(\Delta \alpha \right)⊗q_{ref}\right)v_{I}\\ \approx \left[I_{3\times 3}-\left[\Delta \alpha \times \right]\right]A\left(q_{ref}\right)v_{I}=A\left(q_{ref}\right)v_{I}+\left[A\left(q_{ref}\right)v_{I}\times \right]\Delta \alpha \end{array}$$

*where* Δα *refers to the small attitude error that has components of roll, pitch and yaw error angles for any rotation sequence. As for the MEKF, the reference quaternion used in the measurement update is* ${\hat{q}}_{k|k-1}$ *, also note that the attitude error estimate has been reset to zero in the last time recursion. In this respect, the predicted measurement in MEKF is given by*

(6) $$h_{k}\left({\hat{x}}_{k|k-1}\right)=\left[\begin{array}{c} A\left({\hat{q}}_{k|k-1}\right)r_{1}\\ \vdots \\ A\left({\hat{q}}_{k|k-1}\right)r_{n} \end{array}\right]$$

*As for the SMEKF, the reference quaternion used in the measurement update is the posteriori quaternion estimate after the last vector observation has been processed. Meanwhile, the reset operation is performed implicitly after each processing of the single vector observation. In this respect, the predicted measurement in SMEKF is given by* $A\left({\hat{q}}_{k}\right)r_{j}$ *, with* ${\hat{q}}_{k}$ *denoting the posteriori quaternion estimate after j-1 vector observations have been processed sequentially. This is the reason why the innovation of the SMEKF has a similar form to that of the MEKF, while having a different form to the MMEKF.*

**Remark** **6.** *A main difference between the proposed SMEKF and the traditional SEKF is that the state covariance is not updated in the sequential process. That is, the reset operation in the sequential processing loop does not affect the state covariance. This is because “the reset operation neither increases nor decreases the total information content of the estimate and it merely moves this information from one part of the attitude representation to another” [4]. The simulation study will show that if the covariance is updated in the sequential process loop, which is similar with that in the SEKF, the improvement in the performance of the resulting attitude estimation algorithm is compromised.*

## 4. Simulation Example

**Figure 1:**
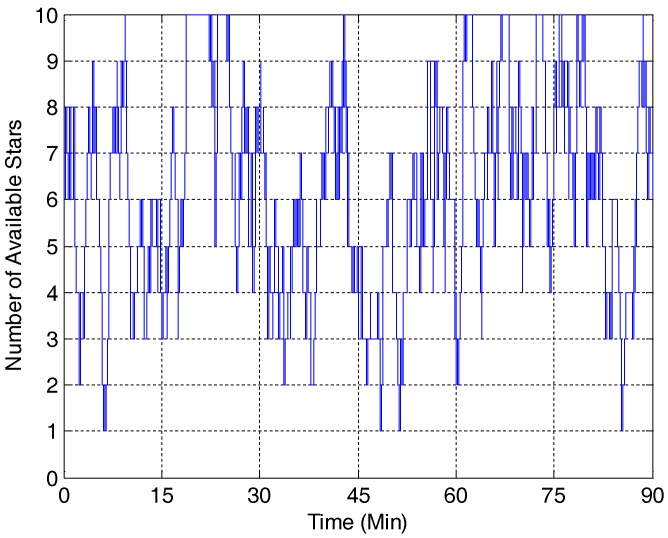
Number of available stars.

In this section, Example 7.2 from [1] (also Example 6.1 from [2]) is used to evaluate the performance of the developed SMEKF against some other attitude estimation methods, including MEKF, MMEKF, SEKF and USQUE. The number of available stars during the simulation is shown in Figure 1. It shows that the star tracker can sense up to 10 stars at one time.

Specifically, several performance comparisons between these algorithms were made using the following four simulation cases:

***Case*** ***1:*** With initial attitude estimation error $\left[1^{\circ };1^{\circ };1^{\circ }\right]$
***Case*** ***2:*** With initial attitude estimation error $\left[30^{\circ };30^{\circ };30^{\circ }\right]$
***Case*** ***3:*** With initial attitude estimation error $\left[-50^{\circ };50^{\circ };160^{\circ }\right]$
***Case*** ***4:*** With initial attitude estimation error $\left[90^{\circ };90^{\circ };180^{\circ }\right]$

The following simulation results are all averaged values from 100 Monte Carlo runs.

**Figure 2:**
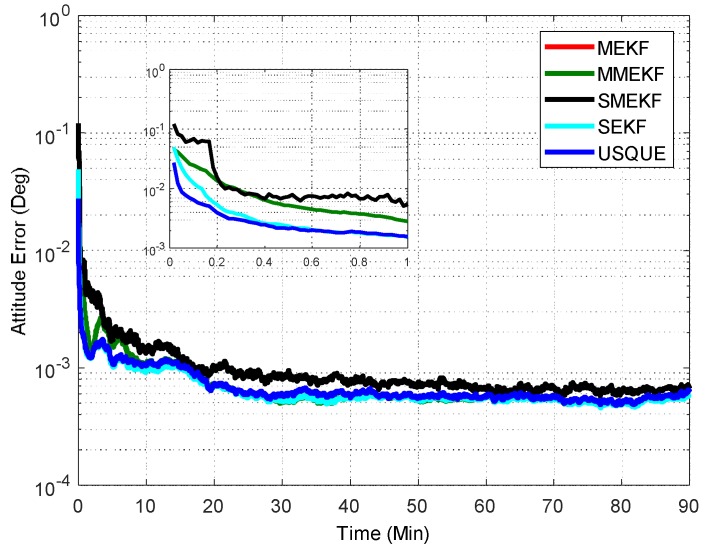
Norm of attitude estimation errors (case 1).

**Figure 3:**
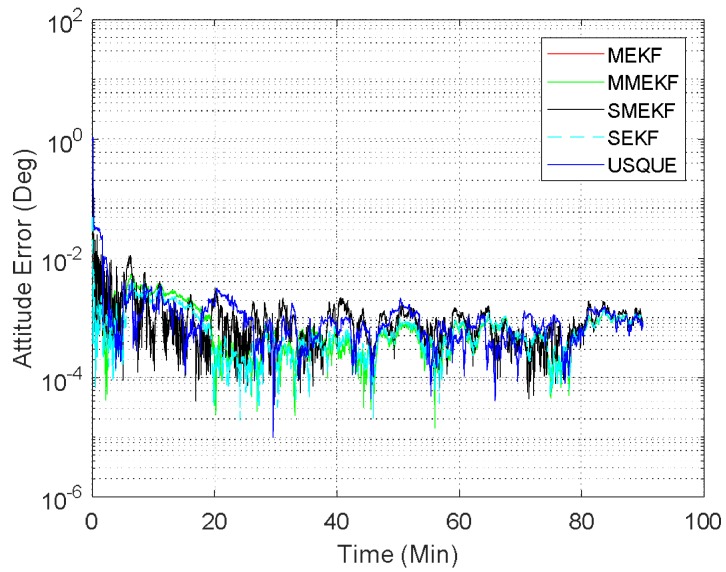
Attitude estimation errors of one Monte Carlo run (case 1).

In the first case, the initial attitude covariance was set to ${\left(1{}^{\circ}\right)}^{2}$ and the bias covariance was set to ${\left(0.2{}^{\circ}/h\right)}^{2}$. The norm of the total attitude estimation error for this case is shown in Figure 2. The performance details from the first minutes of each filter are illustrated in the sub-graph. It shows that all the attitude estimation filters exhibit very similar performance. In fact, the performance of MEKF and MMEKF are identical to each other and their curves cannot be distinguished in Figure 2. The superiority of the SMEKF and USQUE over other filters in terms of accuracy is not so obvious for small initial error conditions. The seemingly underperformance of the proposed SMEKF in Figure 2 may be caused by one contingency of the 100 Monte Carlo runs. We have carried out one time simulation under the initial condition in Case 1. The corresponding results are shown in Figure 3. It is clearly shown that these attitude estimators perform quite similarly.

**Figure 4:**
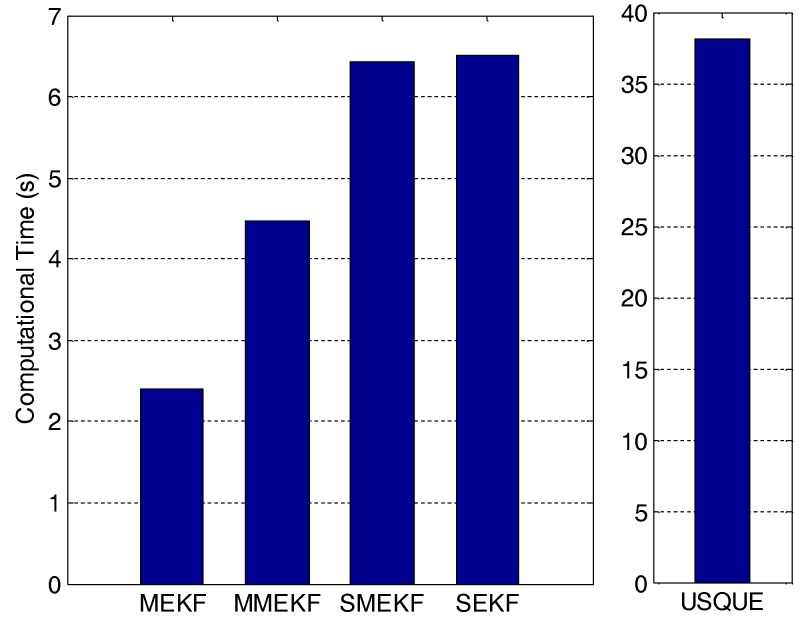
Averaged estimator runtime.

Both algorithms were implemented using Matlab on a computer with 2.66 G CPU, 2.0 G memory and the Windows 7 operating system. The averaged computation times of 100 Monte Carlo runs are shown in Figure 4. Interestingly, the Murrell version does not save computational cost and in contrast, it increases the computational cost. This can be explained as follows: the computational savings from exchanging the inversion of the 3n×3n matrix for the inversion of the 3×3 matrix is only obvious for cases with large numbers of vector observations at a time. For small matrices, lower order components of the computational cost equations can have a large influence on the actual computational cost [14]. The developed SMEKF has an even larger computational burden than the MMEKF. This is because the measurement update and attitude update are both performed for every processing step of the vector observation. The computational cost of the SEKF is slightly greater than that of the SMEKF due to the fact that the covariance is updated in the processing loop of the SEKF. The computational cost of the USQUE is much larger than that of the other filters. In this respect, if other filters show appropriate performance, the USQUE will be not preferred due to its large computational cost.

**Figure 5:**
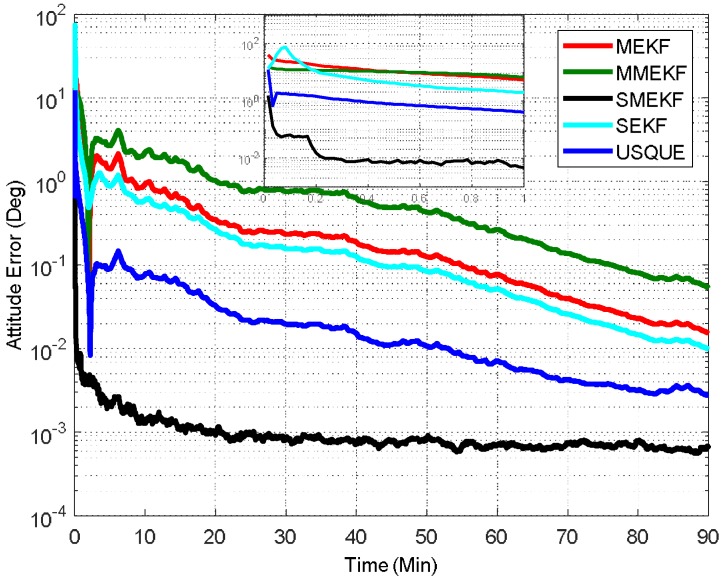
Norm of attitude estimation errors (case 2).

In the second case, the initial attitude covariance was set to ${\left(30{}^{\circ}\right)}^{2}$ and the initial bias covariance was unchanged. The norm of the total attitude estimation error for this case is shown in Figure 5. The performance details for the first minutes of each filter are illustrated in the sub-graph. It shows that the proposed SMEKF still has a fast convergent speed and an accurate steady state. In contrast, the performance of the other four filters was much degraded. In particular, the MMEKF performed even worse than the original MEKF. This indicates that there are no advantages to using MMEKF, as the actual computational savings are also not straightforward. The SEKF can indeed outperform the MEKF, as shown in Figure 5. However, the performance improvement is compromised compared to the SMEKF. This validates that the covariance should not be updated in the sequential processing loop. The USQUE performs much better than the MEKF, which indicates that it is more robust to the large initial estimate error. However, the performance improvement is at the cost of large computational burden, as shown in Figure 4. In this case, the SMEKF was the best, as it had the most accurate performance with appropriate computational cost.

**Figure 6:**
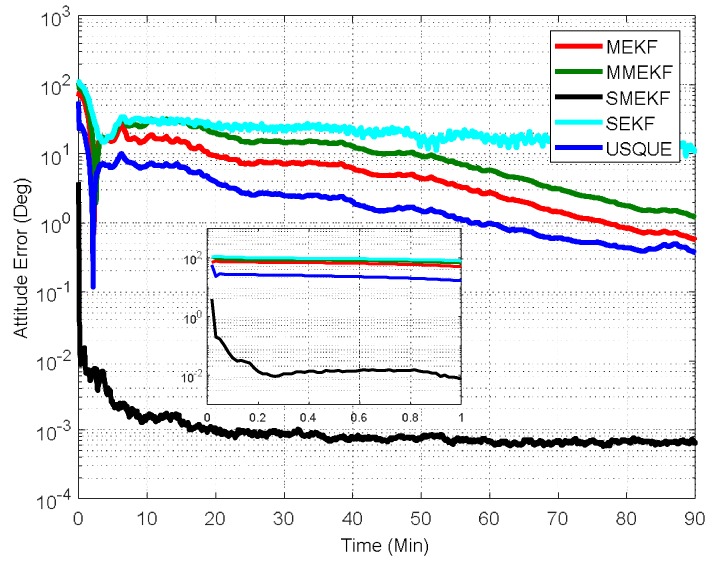
Norm of attitude estimation errors (case 3).

In the third case, the initial attitude covariance was set to ${\left(50{}^{\circ}\right)}^{2}$ and the initial bias covariance is unchanged. This simulation illustrates a possible realistic scenario where the initial attitude estimate is totally unknown. The norm of the total attitude estimation error for this case is shown in Figure 6. The performance details for the first minutes of each filter are illustrated in the sub-graph. In this case, the superiority of the SMEKF over other filters is more obvious. This indicates that the SMEKF can handle the large initial estimate error appropriately and is robust to a wide range of initial estimate error. Interestingly, the SEKF cannot even converge in this case, which indicates that applying the SEKF for the attitude estimate is superficially crude. The performance of the MEKF, MMEKF and USQUE is further degraded. From the three cases, it can be concluded that the robustness of the USQUE is compromised in this simulation example. In contrast, the SMEKF showed consistent performance even in these three cases.

**Figure 7:**
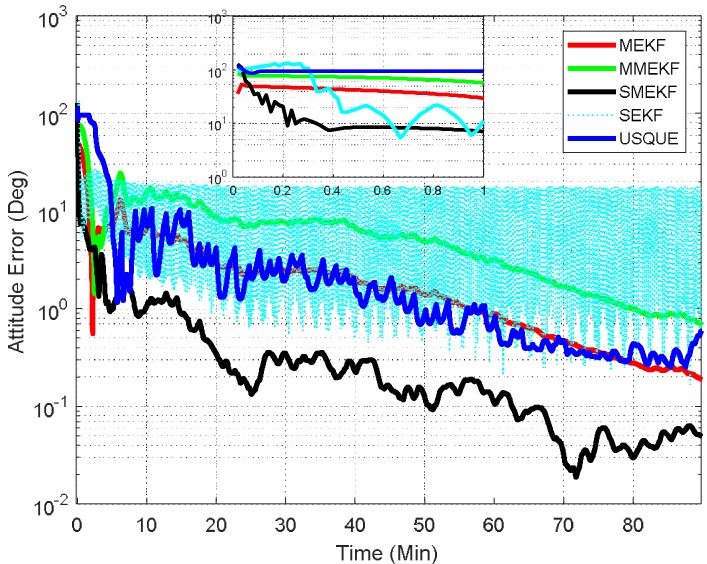
Norm of attitude estimation errors (case 4).

In the fourth case, the initial attitude covariance was set to ${\left(90{}^{\circ}\right)}^{2}$ and the initial bias covariance was unchanged. This case is an extreme condition which may not occur in practice. This case is mainly used to evaluate these attitude estimators under such extreme conditions. The corresponding results are shown in Figure 7. The performance details for the first minutes of each filter are illustrated in the sub-graph. It can be seen that in this case all these attitude estimators cannot converge to the steady-state as compared with the results in the last three cases. However, the superiority of the proposed SMEKF over other attitude estimators can still be observed in this case.

## 5. Conclusions

In this paper, the sequential implementation of the MEKF for attitude estimation using vector observations was developed. In the developed SMEKF, the measurement update and attitude update are performed together for each vector attitude observation individually, providing a more accurate state estimate, for which the linearization of the next vector attitude measurement equation is performed. Meanwhile, the covariance is not updated in the sequential processing loop due to the special characteristics of the reset operation. The spacecraft attitude estimation application demonstrated that the SMEKF can outperform the MEKF and USQUE. It showed quite consistent and accurate performance for a wide range of initial estimate error. Moreover, the computational burden of the developed SMEKF is appropriate for real-time applications.

## Figures and Tables

**Table 1:** Multiplicative extended Kalman filter (MEKF) vs. Murrell’s variation of the MEKF (MMEKF).

**Initialization**$q\left(t_{0}\right)={\hat{q}}_{0}$, $\beta \left(t_{0}\right)={\hat{\beta }}_{0}$, $P\left(t_{0}\right)=P_{0}$
**Time update**
$\hat{\omega }\left(t\right)=\tilde{\omega }\left(t\right)-\hat{\beta }\left(t\right)$
$\dot{\hat{q}}\left(t\right)=\frac{1}{2}\Xi \left(\hat{q}\left(t\right)\right)\hat{\omega }\left(t\right)$
$\dot{P}\left(t\right)=F\left(\hat{x}\left(t\right),t\right)P\left(t\right)+P\left(t\right)F^{T}\left(\hat{x}\left(t\right),t\right)+G\left(t\right)Q_{k-1}G^{T}\left(t\right)$
$F\left(\hat{x}\left(t\right),t\right)=\left[\begin{array}{cc} -\left[\hat{\omega }\left(t\right)\right] & -I_{3\times 3}\\ 0_{3\times 3} & 0_{3\times 3} \end{array}\right]$, $G\left(t\right)=\left[\begin{array}{cc} -I_{3\times 3} & 0_{3\times 3}\\ 0_{3\times 3} & I_{3\times 3} \end{array}\right]$
**Measurement update**
**MEKF**
$h_{k}\left({\hat{x}}_{k|k-1}\right)=\left[\begin{array}{c} A\left({\hat{q}}_{k|k-1}\right)r_{1}\\ \vdots \\ A\left({\hat{q}}_{k|k-1}\right)r_{n} \end{array}\right]$, $H_{k}^{}\left({\hat{x}}_{k|k-1}\right)=\left[\begin{array}{cc} A\left({\hat{q}}_{k|k-1}\right)r_{1} & 0_{3\times 3}\\ \vdots & \vdots \\ A\left({\hat{q}}_{k|k-1}\right)r_{n} & 0_{3\times 3} \end{array}\right]$
$K_{k}=P_{k|k-1}H_{k}^{T}\left({\hat{x}}_{k|k-1}\right){\left[H_{k}^{}\left({\hat{x}}_{k|k-1}\right)P_{k|k-1}H_{k}^{T}\left({\hat{x}}_{k|k-1}\right)+R_{k}\right]}^{-1}$
$P_{k}=\left[I_{6\times 6}-K_{k}H_{k}^{}\left({\hat{x}}_{k|k-1}\right)\right]P_{k|k-1}$
$\Delta {\hat{x}}_{k}=K_{k}\left[{\tilde{y}}_{k}-h_{k}^{}\left({\hat{x}}_{k|k-1}\right)\right]$
**MMEKF**
$\Delta {\hat{x}}_{k}=0_{6\times 1}$,$P_{k}=P_{k|k-1}$
*for* j=1:n
$H_{k,j}^{}\left({\hat{x}}_{k|k-1}\right)=\left[\begin{array}{cc} A\left({\hat{q}}_{k|k-1}\right)r_{j} & 0_{3\times 3} \end{array}\right]$
$K_{k,j}=P_{k}H_{k,j}^{T}\left({\hat{x}}_{k|k-1}\right){\left[H_{k,j}^{}\left({\hat{x}}_{k|k-1}\right)P_{k}H_{k,j}^{T}\left({\hat{x}}_{k|k-1}\right)+\sigma_{j}^{2}I_{3\times 3}\right]}^{-1}$
$P_{k}=\left[I_{6\times 6}-K_{k,j}H_{k,j}^{}\left({\hat{x}}_{k|k-1}\right)\right]P_{k}$
$\Delta {\hat{x}}_{k}=\Delta {\hat{x}}_{k}+K_{k,j}\left[{\tilde{y}}_{k,j}-A\left({\hat{q}}_{k|k-1}\right)r_{j}-H_{k,j}^{}\left({\hat{x}}_{k|k-1}\right)\Delta {\hat{x}}_{k}\right]$
*end*
**Attitude update**
$\Delta {\hat{x}}_{k}=\left[\begin{array}{cc} \Delta {\hat{\alpha }}_{k}^{T} & \Delta {\hat{\beta }}_{k}^{T} \end{array}\right]$
${\hat{q}}_{k}={\hat{q}}_{k|k-1}+0.5\Xi \left({\hat{q}}_{k|k-1}\right)\Delta {\hat{\alpha }}_{k}$
${\hat{\beta }}_{k}={\hat{\beta }}_{k|k-1}+\Delta {\hat{\beta }}_{k}$

**Table 2:** Sequential MEKF (SMEKF).

**Initialization**$q\left(t_{0}\right)={\hat{q}}_{0}$,$\beta \left(t_{0}\right)={\hat{\beta }}_{0}$,$P\left(t_{0}\right)=P_{0}$
**Time update**
$\hat{\omega }\left(t\right)=\tilde{\omega }\left(t\right)-\hat{\beta }\left(t\right)$
$\dot{\hat{q}}\left(t\right)=\frac{1}{2}\Xi \left(\hat{q}\left(t\right)\right)\hat{\omega }\left(t\right)$
$\dot{P}\left(t\right)=F\left(\hat{x}\left(t\right),t\right)P\left(t\right)+P\left(t\right)F^{T}\left(\hat{x}\left(t\right),t\right)+G\left(t\right)Q_{k-1}G^{T}\left(t\right)$
$F\left(\hat{x}\left(t\right),t\right)=\left[\begin{array}{cc} -\left[\hat{\omega }\left(t\right)\right] & -I_{3\times 3}\\ 0_{3\times 3} & 0_{3\times 3} \end{array}\right]$, $G\left(t\right)=\left[\begin{array}{cc} -I_{3\times 3} & 0_{3\times 3}\\ 0_{3\times 3} & I_{3\times 3} \end{array}\right]$
**Measurement update and attitude update**
${\hat{q}}_{k}={\hat{q}}_{k|k-1}$, ${\hat{\beta }}_{k}={\hat{\beta }}_{k|k-1}$
*for* j=1:n
$H_{k,j}^{}\left({\hat{x}}_{k}\right)=\left[\begin{array}{cc} A\left({\hat{q}}_{k}\right)r_{j} & 0_{3\times 3} \end{array}\right]$
$K_{k,j}=P_{k|k-1}H_{k,j}^{T}\left({\hat{x}}_{k}\right){\left[H_{k,j}^{}\left({\hat{x}}_{k}\right)P_{k|k-1}H_{k,j}^{T}\left({\hat{x}}_{k}\right)+\sigma_{j}^{2}I_{3\times 3}\right]}^{-1}$
$\Delta {\hat{x}}_{k}=K_{k,j}\left[{\tilde{y}}_{k,j}-A\left({\hat{q}}_{k}\right)r_{j}\right]$
$\Delta {\hat{x}}_{k}=\left[\begin{array}{cc} \Delta {\hat{\alpha }}_{k}^{T} & \Delta {\hat{\beta }}_{k}^{T} \end{array}\right]$
${\hat{q}}_{k}={\hat{q}}_{k}+0.5\Xi \left({\hat{q}}_{k}\right)\Delta {\hat{\alpha }}_{k}$
${\hat{\beta }}_{k}={\hat{\beta }}_{k}+\Delta {\hat{\beta }}_{k}$
*end*
$P_{k}=\left[I_{6\times 6}-K_{k,n}H_{k,n}^{}\left({\hat{x}}_{k}\right)\right]P_{k|k-1}$
